# Erratum to: “Anxiety and neurometabolite levels in the hippocampus and amygdala after prolonged exposure to predator-scent stress”

**DOI:** 10.18699/VJ20.601

**Published:** 2020-02

**Authors:** O.B. Shevelev, V.E. Tseilikman, N.V. Khotskin, A.S. Khotskina, G.V. Kontsevaya, M.S. Lapshin, M.P. Moshkin, M.V. Komelkovav, I.V. Feklicheva, O.B. Tseilikman, E.B. Manukhina, H.F. Downey, E.L. Zavjalov

**Keywords:** 10.18699/VJ19.528, 10.18699/VJ19.528

## Abstract

Vavilovskii Zhurnal Genetiki i Selektsii = Vavilov Journal of Genetics and Breeding. 2019;23(5):582-587 (in Russian)

Page 587, in Acknowledgements instead of

The animals and behavioral testing are supported by the budget project (No. 0324-2019-0041). The MRI study is supported by the budget project (No. 0259-2019-0004). All studies are implemented using the equipment of Center for Genetic Resources of Laboratory Animals at ICG SB RAS, supported by the Ministry of Education and Science of Russia (Unique ID# of the project: RFMEFI62117X0015).

should read

The animals and behavioral testing are supported by the budget project (No. 0324-2019-0041). The MRI study is supported by the budget project (No. 0259-2019-0004). All studies are implemented using the equipment of Center for Genetic Resources of Laboratory Animals at ICG SB RAS, supported by the Ministry of Education and Science of Russia (Unique ID# of the project: RFMEFI62117X0015). The study was conducted within the basic part of the state task of the Ministry of Science and Higher Education of the Russian Federation (No. 17.7255.2017/8.9).

The original article can be found under DOI 10.18699/VJ19.528

## Abstract (ru)

Вавиловский журнал генетики и селекции. 2019;23(5):582-587

На странице 587 в разделе Благодарности вместо «Работа с животными и поведенческое тестирование выполнены в рамках бюджетного проекта № 0324-2019-0041. Томографические данные получены на средства бюджетного проекта № 0259-2019-0004. Исследование проведено с использованием оборудования ЦКП «Центр генетических ресурсов лабораторных животных» ФИЦ ИЦиГ СО РАН, поддержанного Минобрнауки России (уникальный идентификатор проекта RFMEFI62117X0015)».

следует читать: «Работа с животными и поведенческое тестирование выполнены в рамках бюджетного проекта № 0324-2019-0041. Томографические данные получены на средства бюджетного проекта № 0259-2019-0004. Исследование проведено с использованием оборудования ЦКП «Центр генетических ресурсов лабораторных животных» ФИЦ ИЦиГ СО РАН, поддержанного Минобрнауки России (уникальный идентификатор проекта RFMEFI62117X0015). Исследование выполнено в рамках базовой части государственного задания Минобрнауки России (№ 17.7255.2017/8.9)».

Оригинальная статья может быть найдена по DOI 10.18699/VJ19.528

